# Editorial: Causative mechanism and potential pharmacological and non-pharmacological interventions in sepsis-associated encephalopathy

**DOI:** 10.3389/fneur.2023.1199840

**Published:** 2023-05-16

**Authors:** Yihui Li, You Shang, Jianfeng Xie, Huaibo Zhang, Hui Han, Hao Wang

**Affiliations:** ^1^Department of Critical Care Medicine, Qilu Hospital, Shandong University, Jinan, China; ^2^Department of Critical Care Medicine, Tongji Medical College, Union Hospital, Huazhong University of Science and Technology, Wuhan, China; ^3^Jiangsu Provincial Key Laboratory of Critical Care Medicine, Department of Critical Care Medicine, School of Medicine Southeast University, Zhongda Hospital, Nanjing, China; ^4^Center for Alcohol Research in Epigenetics, Department of Psychiatry, University of Illinois at Chicago, Chicago, IL, United States

**Keywords:** sepsis-associated encephalopathy (SAE), special populations, immune imbalance, microbiota-gut-brain axis, Treg cell

Sepsis is a life-threatening syndrome characterized by multi-organ dysfunction induced by a dysregulated host response to infection (1, 2). Sepsis-associated encephalopathy (SAE) is a widespread dysfunction of the brain that arises as a key complication of sepsis. The incidence of SAE is more than 70% in patients experiencing septic shock, with higher rates observed among elderly individuals, neonates, and those with chronic illnesses. It is associated with long-term neurological damage, including anxiety, memory impairment, and alterations in consciousness. Studies have reported a significantly higher mortality rate among septic patients with SAE (3–5).

A lack of objective and uniform indicators and diagnostic criteria, as well as the neglect of clinical researchers, have resulted in a dearth of epidemiological data on SAE in various populations, including incidence, mortality, risk factors, clinical outcomes, and social and economic burden. Furthermore, there are no animal models fully mimicking the characteristics of SAE (3, 4). Additional investigations are necessary to understand the underlying causative mechanisms of SAE and explore potential pharmacological and non-pharmacological interventions. The goal of this Research Topic is to expand the knowledge of SAE, with a focus on the role of immune disorders. The current issue comprises six articles covering the mechanisms and potential interventions for SAE, utilizing various approaches such as clinical cohort studies, animal models, and microbiome analyses.

## SAE in the elderly and pediatric populations

The epidemiological data on SAE in ICU has consistently lacked on two special populations at both ends of the age spectrum: the elderly and children (6). This topic, for the first time, focuses on the clinical data of SAE in these two special populations. Zhao et al. develop a nomogram to predict SAE in elderly patients, while Chen et al. identify risk factors for SAE in children, which can present with unique symptoms. Depending on the different diagnostic criteria, sepsis affects 10% to over 70% of adult ICU patients (5). Elderly and pediatric populations are not spared from SAE. Zhao et al. review data from 22,361 elderly patients (age ≥ 65 years) in the Medical Information Mart for Intensive Care (MIMIC)-IV database and find that 290 patients (37.1%) had SAE. The in-hospital mortality rate was higher in the SAE group than in the non-SAE group (18.8 vs. 8.9%, *p* < 0.001). They develop a nomogram integrating age, sodium, Sequential Organ Failure Assessment (SOFA) score, heart rate, and body temperature to predict the occurrence of SAE in the elderly population. Chen et al. focus on another special group of sepsis patients, children. In a cohort of 210 children, 91 (43.33%) were diagnosed with SAE. Procalcitonin, calcium, septic shock, Pediatric Logistic Organ Dysfunction-2 (PELOD-2), and midazolam were identified as independent risk factors for SAE, while fentanyl was a protective factor. SAE in children presents unique symptoms, such as anterior fontanelle full/bulging/high tension, muscle hypertonia, muscle hypotonia, hyperreflexia, focal seizure, and generalized seizure.

## Immune imbalance in SAE

Liu et al. and Gao et al. bring more cumulative evidence to this topic supporting dysregulated immune response possibly having a central role in SAE. The pathogenesis of SAE involves a dysregulated immune system in the central nervous system (CNS), disruption of the blood-brain barrier, mitochondrial dysfunction, oxidative stress, and other factors (4, 7). Liu et al. summarize the interactions between the brain and peripheral immune system in sepsis. They propose that SAE is mainly caused by neuronal ischemia, cell death, and the disruption of the “neurocentral-endocrine-immune” networks due to the over-activation of immune cells in the brain mediated by the neutrophils, microglia, astrocytes, and the hypothalamic-pituitary-adrenal axis.

Tregs are important anti-inflammatory cells, and Gao et al. emphasize the regulatory roles of Tregs in neurotransmitter modulation, inflammation resolution, regulation of glial cells, brain endothelial cells, and the brain-gut axis in the pathogenesis of SAE. They propose that adoptive cell transfer therapies with Tregs or derived small extracellular vesicles may be a potential treatment strategy for SAE in the future.

## Microbiota-gut-brain axis drives SAE

The researchers within this topic have brought new perspectives to the mechanisms of SAE: the gut microbiota regulates normal brain function and mediates SAE, and suggests strategies such as probiotics, fecal microbiota transplantation, vagus nerve stimulation, and short-chain fatty acids (SCFAs) might be proposed to treat SAE by modulating the microbiota-gut-brain axis. The gut is the engine of sepsis, and the microbiota-gut-brain axis regulates normal brain function and mediates SAE (8). Barlow et al. summarize the changes in gut microbiota and metabolites in sepsis, including the production of gamma-aminobutyric acid (GABA) by endogenous gut microbiota such as *Bifidobacterium* and *Lactobacillus*. They also review the use of probiotics, fecal microbiota transplantation, and vagus nerve stimulation as strategies to regulate the microbiota-gut-brain axis and treat SAE.

Liao et al. propose another approach to modulate the microbiota-gut-brain axis: SCFAs produced by gut microbiota fermentation of indigestible dietary fiber. They find that the levels of acetate and propionate were significantly reduced in SAE mice, accompanied by gut microbiota dysbiosis, particularly a decrease in SCFA-producing bacteria. SCFAs can antagonize neuroinflammation and protect mouse cognition through G-protein-coupled receptor 43 (GPR43) in SAE.

## Perspectives

In summary, sepsis is a life-threatening systemic disease. In terms of organs, the brain is not a survivor. Among the population, the elderly and children are not exempt from SAE. The gut microbiota-mediated immune dysregulation in sepsis may be an important pathological mechanism of SAE. Moreover, Treg immune cells, gut microbiota, and their metabolites may become promising therapeutic strategies for SAE. Nonetheless, there remain several challenges regarding SAE that require increased attention, as highlighted by the Surviving Sepsis Campaign project. The following recommendations are offered as guidelines for preclinical and clinical investigations:

1) Develop clinically efficient, concise, and easy-to-use diagnostic procedures and prognostic models for SAE by integrating multi-scale measures and analyses to better comprehend the underlying mechanisms of disease progression.
2) Establish animal models of SAE that accurately and comprehensively simulate the condition in humans, rather than relying on simple translations of conventional sepsis models. Additionally, the behavioral experiments in these animal models should be implementable without complex instruments or apparatuses.
3) Explore cost-effective and feasible technologies and drugs that can improve the performance of high-risk SAE patients, ideally while also enhancing their ultimate prognoses.
4) Establish interdisciplinary research teams that include critical care physicians, neurologists, internists, and neuroscientists to break down the barriers between bench and bedside, assist clinical practitioners in understanding the pathophysiological characteristics and research progress of SAE, and help neurologists and critical care scientists uncover more potential mechanisms of the disease.

## Author contributions

All authors listed have made a substantial, direct, and intellectual contribution to the work and approved it for publication.
